# Daily academic and sport-related strain and mental-health-related states among university students in sport-related majors: a 14-day ecological momentary assessment network study

**DOI:** 10.3389/fpsyg.2026.1889946

**Published:** 2026-09-07

**Authors:** Aozhou Wang, Can Ao, Nongqin Guan, Cong Ma

**Affiliations:** 1 Department of Physical Education, Sangji University, Wonju, Republic of Korea; 2 Sports Department, Chongqing Jiaotong University, Chongqing, China; 3 Sports Teaching and Research Group, Chongqing Jiangjin Middle School, Chongqing, China; 4 Department of Physical Education, Namseoul University, Cheonan, Republic of Korea

**Keywords:** anxiety/irritability, dynamic network analysis, ecological momentary assessment, rumination, sleep

## Abstract

University students enrolled in sport-related majors may simultaneously manage academic requirements and regular training or competition, yet the short-term organization of their physical, academic/performance-related, affective, and cognitive states remains insufficiently understood. This study used a 14-day ecological momentary assessment (EMA) design with three smartphone assessments per day and multilevel vector autoregressive modeling. The original recruitment pool included 72 students from one university in Chongqing, China. Five participants were excluded for repeated responses outside the designated EMA windows, and seven were excluded for broader protocol or response-quality failures, including discontinuation of EMA completion and/or repeated invariant, non-informative response patterns. The retained analytic sample comprised 60 students and a balanced panel of 2,520 observations. Eight brief, study-specific momentary indicators were modeled: fatigue, soreness/bodily pain, academic stress, performance worry, depressed mood, tension/anxiety/irritability, rumination, and concentration difficulty. The revised primary temporal model used population-level fixed lagged slopes and showed no singularity or convergence problems. Soreness and fatigue showed the largest temporal out-strength, whereas the tension/anxiety/irritability indicator showed the largest temporal in-strength. In the contemporaneous network, the tension/anxiety/irritability indicator and rumination showed the highest expected-influence and bridge-centrality estimates. Participant-level bootstrap and case-dropping analyses indicated high centrality stability within the retained sample, although between-person edge estimates were substantially less precise. Sensitivity analyses showed that the broad outgoing and contemporaneous structure was comparatively robust to measured training, academic, and time-of-day context, whereas several incoming temporal relations, particularly those involving academic stress, were context-sensitive. Poorer-than-usual sleep remained prospectively associated with higher next-morning levels of all eight states after adjustment for participants' mean sleep, the previous-evening value of the same outcome, study day, and participant-level clustering. These findings characterize conditional associations among the specific momentary indicators measured in this single-university sample; they do not establish causal pathways, comprehensive dual-career functioning, or intervention targets.

## 1 Introduction

University students enrolled in sport-related majors may need to coordinate academic requirements with regular training, competition, and recovery. This overlap is relevant to dual-career frameworks, which emphasize the coordination of educational and sport-related demands (Stambulova and Wylleman, 2015; Stambulova et al., 2024). Existing work has shown that young athletes and college-based sport participants can experience depressive symptoms, anxiety, burnout, sleep difficulties, and barriers to help-seeking, although prevalence estimates vary across contexts, performance levels, and sampling frames (Rice et al., 2016; Wolanin et al., 2016; Edwards et al., 2023; Chang et al., 2020; Aalto et al., 2024). The present study, however, did not operationalize dual-career functioning as a comprehensive multidimensional construct. Instead, it examined a narrower set of momentary subjective states that may arise in a context where university study and sport participation coexist.

A central limitation of the literature is that much of the available evidence remains cross-sectional, retrospective, or service-based. Those approaches are informative for prevalence, care access, and between-person differences, but they are less well-suited to short-term psychological dynamics (Moreland et al., 2018; Young et al., 2023; Shiffman et al., 2008; Myin-Germeys et al., 2018). In everyday university settings, bodily strain, performance worry, poor sleep, academic stress, affective arousal, rumination, and concentration difficulty may become temporally coupled across the day. Such short-term coupling is difficult to recover accurately from global retrospective reports.

Ecological momentary assessment (EMA) is well-suited to this problem because it samples experience close to when it occurs and thereby reduces dependence on retrospective reconstruction (Shiffman et al., 2008; Myin-Germeys et al., 2018). Network approaches provide a complementary framework for describing conditional relations among multiple stress- and mental-health-related states (Borsboom, 2017; Fried et al., 2017; Robinaugh et al., 2020; Bringmann et al., 2022). In intensive longitudinal data, multilevel network models can distinguish within-person lagged relations, within-person same-window conditional associations, and between-person associations among mean levels. These layers are conceptually distinct and should not be interpreted interchangeably.

The present study, therefore, focused on eight repeatedly assessed momentary indicators spanning four theory-informed domains: Physical Burden (fatigue and soreness/bodily pain), Academic/Performance Strain (academic stress and performance worry), Affective Distress (depressed mood and a combined tension/anxiety/irritability indicator), and Cognitive Difficulty (rumination and concentration difficulty). The dual-career literature serves as a contextual framework for understanding why academic and sport-related demands may coexist in this population; the measured indicators should not be treated as a comprehensive assessment of dual-career functioning, objective academic workload, training load, or competition density.

Against this background, the study had three primary aims. First, we examined whether the focal indicators showed sufficient within-person variation to support intensive longitudinal modeling. Second, we estimated temporal, contemporaneous, and between-person networks to characterize their conditional organization and node-level structural positions. Third, we examined whether poorer-than-usual previous-night sleep was prospectively associated with next-morning states after separating within-person sleep fluctuations from between-person sleep differences and adjusting for the previous-evening value of the same outcome. Because all analyses were observational, the goal was to characterize conditional and temporally ordered associations rather than identify causal mechanisms or intervention effects.

## 2 Methods

### 2.1 Participants and design

Participants were recruited through classroom announcements at a university in Chongqing, China. The target population comprised full-time university students enrolled in sport-related majors who were actively engaged in regular training and/or competition during the study period. Participation was voluntary and was not linked to course grades, monetary compensation, or other academic benefits.

The original recruitment pool included 72 students. Twelve participants were excluded during the initial protocol and response-quality screening before construction of the primary analytic panel. Five were excluded because of repeated responses outside the designated EMA windows. The remaining seven failed broader protocol or response-quality criteria, including discontinuation of EMA completion and/or repeated invariant, non-informative response patterns across the focal EMA items. Such patterns included, for example, repeatedly assigning the same extreme value across the full set of focal items over multiple prompts rather than providing differentiated momentary reports. A score of zero was itself a valid response; exclusion concerned repeated response patterns judged non-informative in combination with protocol-quality screening rather than any single response value. Participant-level exclusion was used to obtain a temporally aligned panel with complete coverage of the scheduled assessments. The final analytic sample therefore comprised 60 students (35 men and 25 women), aged 18–22 years and spanning the first through third years of study, with representation from individual and team sports.

The study used a 14-day EMA design with three scheduled assessments per day (morning, midday, and evening), corresponding to 42 assessments per retained participant. The final primary analytic panel therefore contained 60 × 42 = 2, 520 observations for the eight focal momentary indicators. The retention proportion of 60/72 (83.3%) refers to participant retention and should not be interpreted as the original cohort's prompt-level completion rate.

The dataset retained for the present analyses is the cleaned analytic dataset generated after the initial protocol and response-quality screening. Complete pre-cleaning participant-level raw records for the 12 excluded participants were not preserved in a form that permits retrospective reconstruction of their individual valid and invalid prompts. Consequently, exact prompt-level completion statistics for the original 72-person recruitment pool, formal retained-vs.-excluded participant comparisons, and an all-valid-observation reanalysis including excluded participants cannot now be reconstructed.

The study protocol and use of de-identified participant data were reviewed and approved by the Academic Ethics and Science-and-Technology Ethics Committee of Chongqing Jiaotong University.

### 2.2 Procedure

At study entry, participants completed a baseline form recording demographic and sport-related information. For the retained analytic panel, the 14-day EMA monitoring period ran from October 13 to October 26, 2025. During this period, smartphone prompts were scheduled three times daily within fixed two-hour response windows: 07:00–09:00 (morning), 13:00–15:00 (midday), and 21:00–23:00 (evening). Participants responded using a smartphone slider interface. Responses submitted outside the designated windows were treated as protocol-noncompliant during the initial screening process, and missed core entries were not retroactively backfilled for construction of the retained balanced panel.

Contextual variables were also recorded at each prompt. Training/competition status was categorized as no formal training/recovery-rest, routine training, or competition/selection/high-intensity competitive context. Academic-event status was categorized as no salient academic event, routine academic activity, elevated academic task/preparation demand, or examination/quiz context. These variables represented external situational conditions and were not included as nodes in the primary psychological-state network; they were examined in additional contextual sensitivity analyses.

### 2.3 EMA measures

Eight brief, study-specific momentary indicators were included in the primary network: fatigue, soreness/bodily pain, academic stress, performance worry, depressed mood, tension/anxiety/irritability, rumination, and concentration difficulty. The items were designed for the present intensive EMA protocol to capture psychologically proximal subjective states rather than comprehensive or diagnostic assessments of the corresponding constructs. Because the protocol required three assessments per day for 14 consecutive days, brief single-item indicators were used to reduce repeated-measure burden and preserve sensitivity to momentary fluctuation. These items were not treated as validated single-item substitutes for multidimensional clinical scales.

The instrument was administered in Chinese. At each Q1–Q8 assessment, participants were instructed to rate their true experience “right now” using a smartphone slider ranging from 0 (“not at all”) to 100 (“extremely strong”). The items assessed current fatigue, muscle soreness/bodily pain, academic stress, training/competition worry, depressed mood, tension/anxiety/irritability, repetitive negative thinking, and concentration difficulty. The item abbreviated as “Anxiety/Irritability” in the analyses asked whether participants felt tense, anxious, or irritable at that moment and should therefore be interpreted as a broad indicator of negative affective arousal rather than as a specific clinical measure of anxiety or irritability.

Previous-night sleep was assessed only at the morning prompt using the statement “My sleep quality last night was poor,” rated from 0 (“completely disagree”) to 100 (“completely agree”), such that higher scores indicated poorer perceived sleep quality. The complete original Chinese wording, English translations, response anchors, assessment frequency, and analytic labels for all EMA items are provided in Supplementary Table S1. The assessment structure and analytic use of the study variables are summarized in Table 1.

**Table 1:** EMA assessment structure and analytic use of the study variables.

Code	Analytic label	Assessment frequency	Analytic use
Q1	Fatigue	Morning, midday, and evening	Primary multilevel network
Q2	Soreness/bodily pain	Morning, midday, and evening	Primary multilevel network
Q3	Academic stress	Morning, midday, and evening	Primary multilevel network
Q4	Performance worry	Morning, midday, and evening	Primary multilevel network
Q5	Depressed mood	Morning, midday, and evening	Primary multilevel network
Q6	Anxiety/irritability (tension/anxiety/irritability item)	Morning, midday, and evening	Primary multilevel network
Q7	Rumination	Morning, midday, and evening	Primary multilevel network
Q8	Concentration difficulty	Morning, midday, and evening	Primary multilevel network
Q9	Poor sleep (previous night)	Morning only	Additional mixed-effects sleep models

Q1–Q8 used 0–100 current-intensity ratings; Q9 used 0–100 agreement anchors, with higher values indicating poorer previous-night sleep. Exact wording and anchors are reported in Supplementary Table S1.

### 2.4 Analytic strategy

Descriptive statistics and intraclass correlation coefficients (ICCs) were first calculated to characterize mean levels and the relative balance between within-person and between-person variability.

The eight repeatedly assessed indicators were then entered into a multilevel vector autoregressive model using the mlVAR package (version 0.6.1) with the lmer estimator (Epskamp et al., 2026). The revised primary model used one lag, population-level fixed temporal slopes (temporal = fixed), an orthogonal contemporaneous specification (contemporaneous = orthogonal), grand-mean standardization (scale = TRUE), and person-mean centering of lagged predictors without additional participant-specific rescaling (scaleWithin = FALSE). Study day and assessment number within day were supplied as the day and beep variables, respectively. Analyses were conducted in R using mlVAR 0.6.1 and lme4.

The temporal network represented within-person conditional lagged associations between successive retained prompts within the same study day. Because study day was supplied as the day variable, evening assessments were not linked to the following morning in the primary temporal model. The observed intervals were approximately 5.92 h from morning to midday and 8.08 h from midday to evening. Directed temporal edges therefore indicate model-based temporal ordering and conditional prediction over successive within-day prompts, not experimentally identified causal effects. The contemporaneous network represented within-person conditional associations among states measured within the same assessment window after accounting for lagged relations. For the contemporaneous and between-person Gaussian graphical-model layers, edge weights reported as *r* are partial correlations rather than zero-order correlations. The between-person network represented conditional associations among participants' mean levels across the 14-day period and should not be used to infer within-person dynamics.

An initially fitted orthogonal random-slope temporal specification produced boundary singular fits for all eight temporal outcome models. The revised fixed-slope temporal specification produced no singular fits or convergence warnings and yielded a virtually identical fixed-effect temporal coefficient matrix (Pearson *r*>0.999, 100% sign agreement). A participant-specific within-person scaling sensitivity analysis also yielded a highly similar temporal matrix (Pearson *r* = 0.989, Spearman ρ = 0.993, 100% sign agreement). Two standardized temporal coefficients exceeded 1 in magnitude in the final specification. Because these estimates are simultaneous multivariable regression coefficients rather than correlations, they are not mathematically restricted to the [−1, 1] interval. Collinearity diagnostics for the person-centered lagged predictors showed a maximum absolute pairwise correlation of 0.83 and a maximum variance inflation factor of 9.97; unusually large coefficients were therefore interpreted cautiously as conditional multivariable associations rather than standalone effect sizes. A classical fixed-effect spectral-radius diagnostic was also inspected descriptively (approximately 1.003 for the revised fixed-slope matrix and 0.868 under participant-specific within-person scaling). Because the present analysis is a finite multilevel EMA model with day boundaries and unequal within-day intervals, this diagnostic was not treated as a formal proof of stationarity; the manuscript therefore does not claim global time-series stationarity.

**Figure 1:**
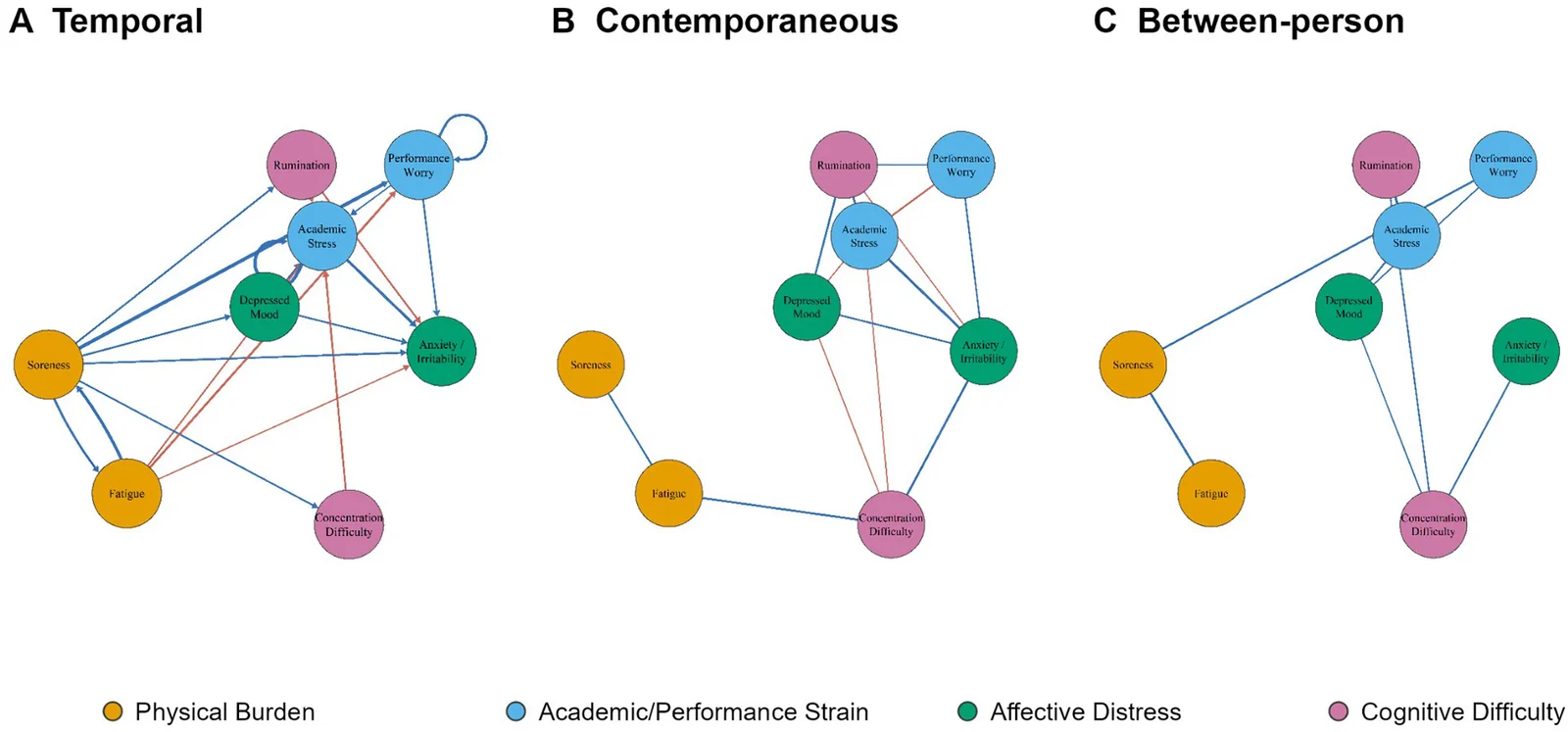
Multi-layer network of measured momentary strain- and mental-health-related states. **(A)** shows the within-person temporal network, representing conditional lag-1 associations between successive retained within-day EMA prompts. **(B)** shows the within-person contemporaneous network, representing conditional associations within the same assessment window after accounting for lagged relations. Panel **(C)** shows the between-person network, representing conditional associations among participant-level mean scores across the 14-day period. Lag-1 in **(A)** denotes successive retained within-day prompts rather than a fixed elapsed-time interval; overnight transitions were excluded from the primary temporal model. Blue edges indicate positive and red edges negative conditional associations, and thicker edges indicate larger absolute coefficients. Node colors denote the four prespecified communities: Physical Burden, Academic/Performance Strain, Affective Distress, and Cognitive Difficulty. For visual clarity only, panel-specific display cutoffs were applied: temporal cross-lagged edges with |β|≥0.34, temporal autoregressive self-loops with |β|≥0.72, contemporaneous edges with absolute weights ≥0.20, and between-person edges with absolute weights ≥.30 are displayed. These cutoffs were not used for model estimation, bootstrap analyses, centrality calculations, or complete coefficient reporting. Temporal arrows indicate model-based temporal ordering and conditional prediction rather than experimentally established causal influence.

Node-level indices were treated as descriptive summaries of structural position. Temporal out-strength and in-strength were calculated from the absolute cross-lagged weights retained in the fixed-effect temporal network, with autoregressive self-loops excluded. Contemporaneous expected influence (EI) and bridge expected influence were calculated from the contemporaneous fixed-effect network. Four communities were specified a priori: Physical Burden (fatigue, soreness), Academic/Performance Strain (academic stress, performance worry), Affective Distress (depressed mood, tension/anxiety/irritability), and Cognitive Difficulty (rumination, concentration difficulty) (Jones et al., 2021). The word “influence” in the formal names Expected Influence and Bridge Expected Influence refers to network-centrality metrics and does not imply causal influence, clinical importance, or intervention effectiveness. Centrality calculations used model-retained edges rather than the stronger display cutoffs used solely to simplify (Figure 1).

Network accuracy and centrality stability were evaluated by participant-level resampling. We performed 1,000 cluster-bootstrap resamples in which participants, rather than individual observations, were sampled with replacement, preserving the within-person EMA structure. Bootstrap distributions were used to obtain 95% intervals for edge weights and centrality indices. Participant-dropping stability was evaluated at deletion proportions from 10% to 75%, with 200 resamples per level. For each subset, temporal out-strength, temporal in-strength, contemporaneous EI, and bridge EI were correlated with the corresponding full-sample estimates. A conservative correlation-stability coefficient was defined as the largest deletion proportion for which at least 95% of all resamples, counting failed fits as not meeting the criterion, retained a correlation of at least 0.70 with the full-sample estimate.

Because the two within-day lag intervals were unequal, transition-specific sensitivity analyses re-estimated the temporal model separately for morning → midday and midday → evening transitions. Evening → next-morning transitions were examined separately as an exploratory comparison because they were excluded from the primary temporal model. Time-of-day differences in mean levels were additionally examined using mixed-effects models including assessment period and study day with participant random intercepts. Elapsed-time distributions and transition-specific results are reported in Supplementary Tables S2–S4.

Contextual indicators of training/competition status and academic events were not included as nodes because they represented situational conditions rather than momentary psychological states. As a sensitivity analysis, each focal EMA indicator was first adjusted for training/competition-status category, academic-event category, assessment period, and study day using mixed-effects models with participant-specific random intercepts. The multilevel network was then re-estimated from these context-adjusted series using the same fixed-temporal specification, and the resulting temporal and contemporaneous matrices and centrality rankings were compared with the primary network. These results are reported in Supplementary Table S5.

Finally, previous-night sleep was decomposed into within-person and between-person components. The within-person component represented the deviation of each morning's poor-sleep score from that participant's own mean poor-sleep score across the monitoring period; the between-person component represented the participant's mean poor-sleep level relative to the sample mean. Each next-morning outcome was modeled as a function of within-person poor sleep, between-person mean poor sleep, the same outcome measured at the previous-evening prompt, and study day, with participant-specific random intercepts. Because a previous-evening baseline was unavailable for Day 1, these prior-outcome-adjusted models used Days 2–14, yielding 780 morning observations from 60 participants. The within-person poor-sleep coefficient was treated as the primary sleep estimate. These models establish temporal ordering but remain observational and are interpreted as prospective within-person associations rather than causal effects.

Complete temporal, contemporaneous, and between-person coefficient sets with participant-level bootstrap 95% intervals are provided in Supplementary Table S6.

## 3 Results

### 3.1 Compliance, descriptive statistics, and within-person variability

The final primary modeling dataset contained 2,520 observations from 60 retained participants, corresponding to complete coverage of the 14-day, three-prompts-per-day schedule within the retained analytic panel. This should not be interpreted as the prompt-level completion rate of the original 72-person recruitment pool, because the complete pre-cleaning records of excluded participants are not reconstructable.

Descriptive statistics and ICCs for the focal indicators are presented in Table 2. Mean levels were highest for concentration difficulty (*M* = 65.35, *SD* = 7.48), academic stress (*M* = 64.97, *SD* = 13.72), rumination (*M* = 64.57, *SD* = 8.86), performance worry (*M* = 64.36, *SD* = 9.61), and fatigue (*M* = 64.12, *SD* = 13.08). Depressed mood showed the lowest mean level (*M* = 48.68, *SD* = 7.66), whereas soreness (*M* = 51.69, *SD* = 12.20) and the tension/anxiety/irritability indicator (*M* = 55.32, *SD* = 8.45) occupied an intermediate range.

**Table 2:** Descriptive statistics and intraclass correlation coefficients (ICCs) for focal indicators.

Indicator	Mean	SD	ICC
Fatigue	64.12	13.08	0.25
Soreness	51.69	12.20	0.50
Academic stress	64.97	13.72	0.22
Performance worry	64.36	9.61	0.18
Depressed mood	48.68	7.66	0.44
Anxiety/irritability	55.32	8.45	0.42
Rumination	64.57	8.86	0.40
Concentration difficulty	65.35	7.48	0.32

ICCs ranged from 0.18 to 0.50, indicating substantial within-person variation across all focal indicators. Performance worry showed the lowest ICC (0.18), whereas soreness showed the highest ICC (0.50), indicating a comparatively larger between-person component for soreness. The ICC pattern supported examination of within-person dynamics while also confirming that the indicators contained non-negligible between-person variation.

### 3.2 Temporal network

**Figure 2:**
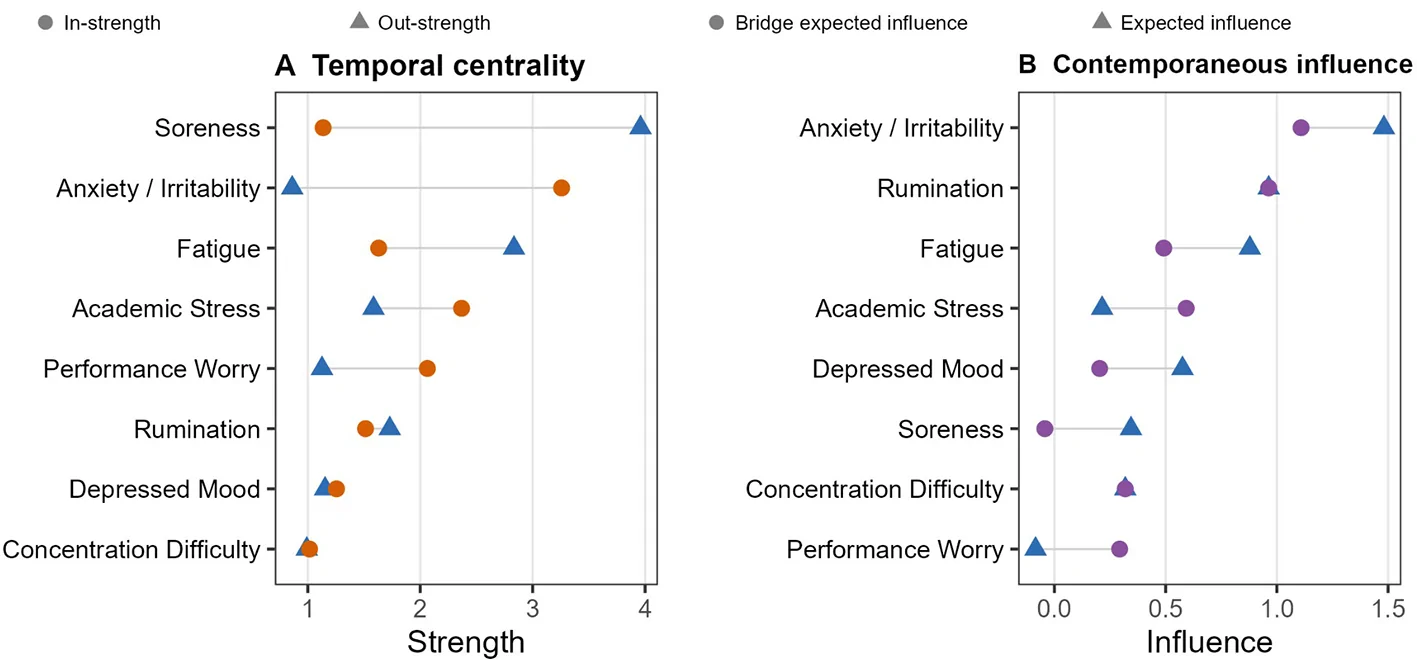
Node-level centrality summaries for the within-person networks. **(A)** shows temporal in-strength and out-strength. **(B)** shows contemporaneous expected influence and bridge expected influence. These indices summarize structural position within the fitted network and should not be interpreted as measures of causal influence, clinical importance, or intervention effectiveness.

Figure 1A shows the revised within-person temporal network, and Figure 2A summarizes temporal out-strength and in-strength. The largest out-strength estimates were observed for soreness (3.96, bootstrap 95% CI [3.50, 4.40]), fatigue (2.83 [2.51, 3.33]), rumination (1.73 [1.56, 1.93]), and academic stress (1.58 [1.43, 1.77]). The largest in-strength estimates were observed for the tension/anxiety/irritability indicator (3.26 [3.01, 3.53]), academic stress (2.37 [2.28, 2.60]), and performance worry (2.06 [1.89, 2.28]). These values describe structural positions in the within-day lagged network rather than causal influence.

Among the larger cross-lagged coefficients were soreness → performance worry (β = 1.17, bootstrap 95% CI [1.08, 1.25]), soreness → fatigue (β = 0.71 [0.65, 0.77]), and academic stress → the tension/anxiety/irritability indicator (β = 0.80 [0.73, 0.88]). A comparatively large negative coefficient was observed for fatigue → performance worry (β = −0.70 [-0.78, -0.63]). These coefficients are conditional on the other lagged predictors in the multivariable model and should not be interpreted as raw pairwise correlations or causal effects.

The final fixed-slope temporal specification showed no singularity or convergence warnings across the eight outcome models. All 1,000 participant-level bootstrap models converged successfully. Of the 56 cross-lagged temporal coefficients, five had bootstrap 95% intervals that included zero. Participant-specific within-person scaling produced a highly similar temporal matrix, supporting the broad robustness of the estimated structure to this scaling choice.

### 3.3 Contemporaneous network

Figure 1B shows the within-person contemporaneous network, and Figure 2B summarizes expected influence and bridge expected influence. The strongest positive contemporaneous partial correlations included academic stress–the tension/anxiety/irritability indicator (*r* = 0.65, bootstrap 95% CI [0.62, 0.67]), the tension/anxiety/irritability indicator–concentration difficulty (*r* = 0.51 [0.46, 0.55]), depressed mood–rumination (*r* = 0.47 [0.42, 0.52]), and fatigue–concentration difficulty (*r* = 0.46). These coefficients represent same-window conditional associations after accounting for lagged relations.

Expected influence was highest for the tension/anxiety/irritability indicator (EI = 1.48, bootstrap 95% CI [1.40, 1.55]), followed by rumination (0.96 [0.87, 1.03]) and fatigue (0.88 [0.78, 0.94]). Bridge expected influence was likewise highest for the tension/anxiety/irritability indicator (1.11 [1.04, 1.18]) and rumination (0.96 [0.89, 1.03]), followed by academic stress (0.59 [0.54, 0.64]) and fatigue (0.49 [0.41, 0.54]). These rankings indicate prominent structural and cross-community positions within the fitted contemporaneous network; they do not identify clinically privileged or causally effective intervention targets.

Bootstrap precision was comparatively high for the contemporaneous layer: three of 28 complete contemporaneous edge intervals included zero.

### 3.4 Between-person network

Figure 1C shows conditional associations among participants' mean levels across the 14-day period. Among the larger positive between-person partial correlations were academic stress—rumination (*r* = 0.74, bootstrap 95% CI [0.59, 0.85]), fatigue—soreness (*r* = 0.68 [0.53, 0.79]), soreness—performance worry (*r* = 0.58), the tension/anxiety/irritability indicator—concentration difficulty (*r* = 0.48), and academic stress—depressed mood (*r* = 0.44). These findings indicate that participants with higher mean levels of one indicator tended to have higher mean levels of the associated indicator across the monitoring period; they do not imply that within-person increases in one state produce within-person changes in another.

The between-person layer was substantially less precise than the two within-person networks: 18 of 28 bootstrap 95% edge intervals included zero. Accordingly, the between-person pattern is treated as descriptive and is interpreted more cautiously than the temporal and contemporaneous networks.

### 3.5 Network accuracy, stability, and sensitivity analyses

All 1,000 participant-level cluster-bootstrap models converged successfully. Participant-dropping analyses indicated high stability of the four reported centrality indices. Using the conservative criterion that failed subset fits counted as not satisfying *r*≥0.70, the correlation-stability coefficient at *r* = 0.70 was 0.70 for temporal out-strength, temporal in-strength, contemporaneous EI, and bridge EI. At 70% participant deletion, 199 of 200 subset models converged, and the fifth-percentile correlations with the full-sample estimates were 0.97 for temporal out-strength, 0.96 for temporal in-strength, 0.97 for contemporaneous EI, and 0.95 for bridge EI. Supplementary Figure S1 presents participant-bootstrap centrality intervals, and Supplementary Figure S2 presents the case-dropping stability curves. At 75% deletion, only 188 of 200 subset models converged (94%); this level therefore did not satisfy the conservative 95% criterion despite high correlations among successfully fitted subsets.

The primary temporal model pooled the two within-day transitions. The observed mean elapsed times were 5.92 h for morning → midday and 8.08 h for midday → evening. Transition-specific temporal matrices correlated *r* = 0.73 and *r* = 0.80, respectively, with the pooled primary matrix, whereas the two transition-specific matrices correlated more moderately with one another (*r* = 0.51). The broad outgoing pattern was replicated: soreness and fatigue remained the leading out-strength nodes in both within-day transition models. Some individual relations were more time-of-day dependent; for example, academic stress → the tension/anxiety/irritability indicator was stronger from midday to evening than from morning to midday. The exploratory evening → next-morning matrix differed markedly from the primary within-day network (*r* = 0.11), supporting the decision not to treat overnight transitions as exchangeable with the primary within-day lag. Mixed-effects models also showed systematic time-of-day differences in mean levels, including higher fatigue and soreness later in the day. Detailed transition and time-of-day results are reported in Supplementary Tables S2–S4.

Measured situational context was also strongly associated with momentary state levels. Relative to no formal training/recovery-rest contexts, routine training was associated with approximately 15.21 points higher fatigue, 13.92 points higher soreness, and 12.95 points higher performance worry, while competition/selection/high-intensity competitive contexts showed still larger differences for these indicators. Examination/quiz contexts were associated with approximately 27.01 points higher academic stress, 11.80 points higher tension/anxiety/irritability, and 9.55 points higher rumination than no salient academic-event contexts.

After adjusting the focal momentary series for training/competition status, academic-event status, assessment period, and study day, the temporal and contemporaneous coefficient matrices remained moderately to highly similar to the primary networks (Pearson *r* = 0.81 and *r* = 0.89, respectively). Temporal out-strength rankings were highly similar (*r* = 0.99), as were contemporaneous EI (*r* = 0.96) and bridge EI (*r* = 0.89). Temporal in-strength was substantially more context-sensitive (*r* = 0.14). Several lagged associations involving academic stress were attenuated after contextual adjustment; for example, academic stress → the tension/anxiety/irritability indicator decreased from approximately 0.80 to 0.18. The results, therefore, support relative robustness of the broad outgoing and contemporaneous structure while indicating that some incoming temporal relations are embedded in training, academic, and time-of-day context. Detailed results are reported in Supplementary Table S5.

### 3.6 Sleep quality and next-morning states

**Figure 3:**
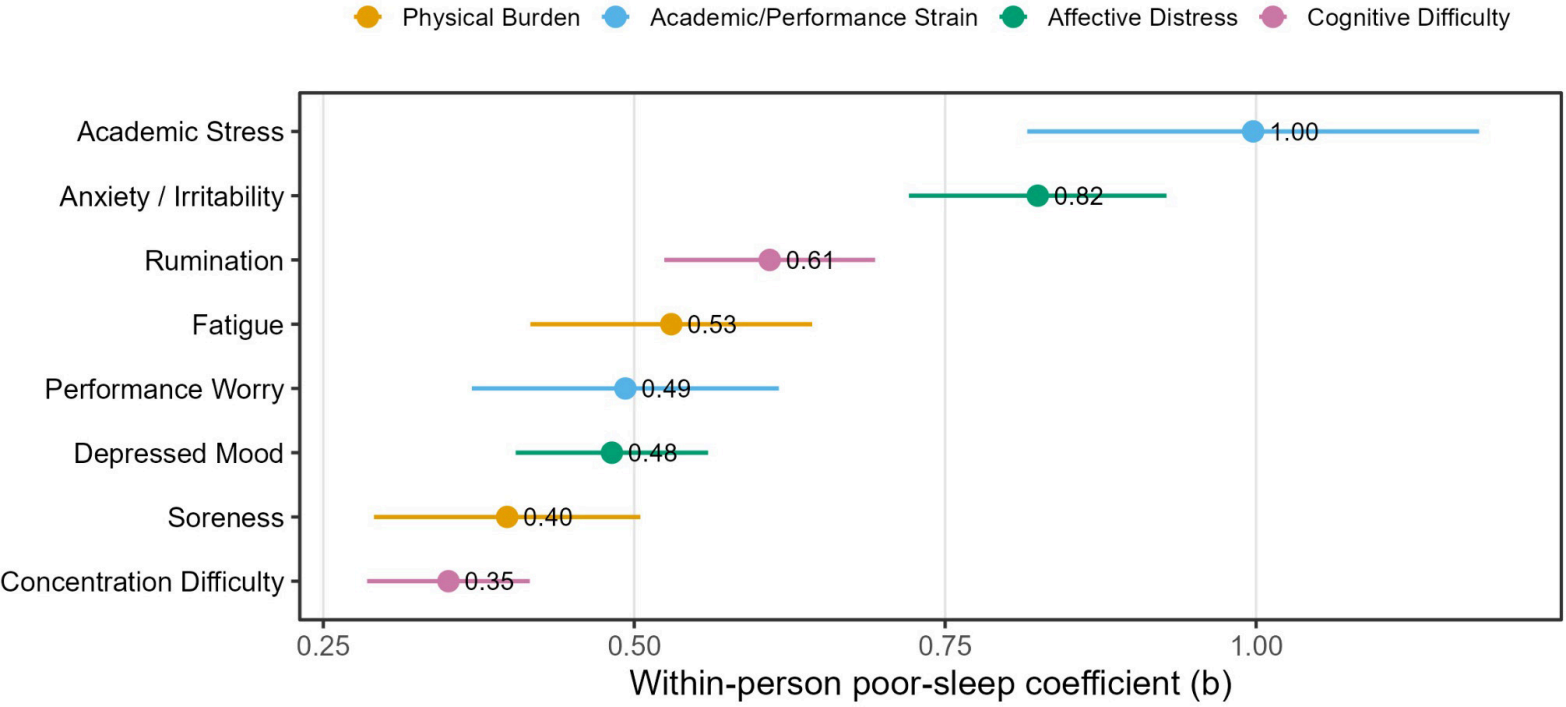
Within-person associations between poorer previous-night sleep and next-morning states. Points represent within-person poor-sleep coefficients from mixed-effects models adjusted for participants' mean poor-sleep level, the previous-evening value of the same outcome, study day, and participant-level random intercepts. Horizontal bars indicate 95% confidence intervals. Higher Q9 scores indicate poorer previous-night sleep quality.

Figure 3 shows the primary within-person poor-sleep coefficients from the revised prior-outcome-adjusted mixed-effects models. Poorer-than-usual sleep was prospectively associated with higher next-morning levels of all eight measured states after adjustment for participants' mean poor-sleep level, the previous-evening value of the same outcome, study day, and participant-level random intercepts.

The largest within-person associations were observed for academic stress (*b* = 1.00, 95% CI [0.82, 1.18]), the tension/anxiety/irritability indicator (*b* = 0.82, [0.72, 0.93]), and rumination (*b* = 0.61, [0.52, 0.69]). Positive associations were also observed for fatigue (*b* = 0.53, [0.42, 0.64]), performance worry (*b* = 0.49, [0.37, 0.62]), depressed mood (*b* = 0.48, [0.40, 0.56]), soreness (*b* = 0.40, [0.29, 0.50]), and concentration difficulty (*b* = 0.35, [0.29, 0.42]); all *p* < 0.001. Each model used 780 observations from 60 participants, and none showed a singular fit.

## 4 Discussion

This study used intensive EMA and multilevel network analysis to characterize the organization of eight brief momentary indicators among students enrolled in sport-related majors at one Chinese university. The results should be understood at the level of the specific indicators measured, not as a complete network of mental-health disorders or dual-career functioning. The three network layers also represent distinct levels of analysis: the temporal and contemporaneous networks describe within-person relations, whereas the between-person network describes conditional associations among 14-day participant means.

At the within-person temporal level, soreness and fatigue occupied the most prominent outgoing structural positions. Higher soreness at one retained within-day prompt was conditionally associated with higher subsequent levels of several states, including performance worry and fatigue, while fatigue also showed substantial outgoing connectivity. These findings support the relevance of subjective bodily burden to the daily organization of the measured states. However, temporal ordering alone does not establish causal influence. The coefficients may reflect unmeasured time-varying factors, reciprocal processes operating at different timescales, or other observational dependencies. The transition-specific analyses also showed that the temporal network should be interpreted as an average within-day structure rather than as a time-invariant process.

Academic stress showed a comparatively prominent outgoing position in the primary temporal network and cross-domain connectivity in the contemporaneous network. At the same time, contextual sensitivity analyses substantially qualified its temporal interpretation. Several incoming relations involving academic stress and the academic stress → tension/anxiety/irritability coefficient were attenuated after adjustment for training, academic-event, and time-of-day conditions. Thus, the temporal position of academic stress appears more context dependent than the outgoing physical-burden pattern. This is consistent with the broader dual-career literature in the limited sense that academic and sport-related demands coexist within students' daily schedules (Stambulova and Wylleman, 2015; Stambulova et al., 2024; Aalto et al., 2024); it should not be interpreted as evidence that the study directly measured a multidimensional dual-career construct.

At the within-person contemporaneous level, the combined tension/anxiety/irritability indicator and rumination showed the highest expected-influence and bridge-centrality estimates. These findings indicate that these indicators were strongly embedded within the same assessment-window conditional network and showed substantial cross-community connectivity. Because Q6 combined tension, anxiety, and irritability in a single item, its structural prominence cannot be attributed specifically to any one of these experiences. Likewise, centrality does not establish that experimentally changing a central node would produce downstream improvement. The results are better viewed as descriptive evidence that may guide monitoring priorities and generate hypotheses for future intervention studies.

The between-person network identified several associations among participants' mean levels, including academic stress–rumination and fatigue–soreness. These associations concern stable individual differences over the 14-day period and should not be translated into within-person mechanisms. Moreover, bootstrap uncertainty was substantially greater for this layer than for the temporal and contemporaneous networks. The between-person findings are therefore best treated as descriptive and hypothesis-generating.

The revised sleep analyses provide a separate prospective within-person result. On nights when participants reported poorer sleep than their own usual level, they tended to report higher next-morning academic stress, tension/anxiety/irritability, rumination, fatigue, performance worry, depressed mood, soreness, and concentration difficulty, even after adjustment for the previous-evening level of the same outcome. This indicates that previous-night sleep provides information about next-morning vulnerability beyond stable between-person sleep differences and the immediately preceding level of the same state. Nevertheless, the observational design does not establish sleep as a causal upstream factor, and unmeasured time-varying variables may contribute to these associations.

From an applied perspective, the structural prominence and stability of several indicators may support more time-sensitive monitoring rather than immediate intervention targeting. In this sample, soreness, academic stress, the combined tension/anxiety/irritability indicator, rumination, and previous-night sleep may be useful candidate indicators for closer monitoring. Centrality alone, however, is not a sufficient basis for intervention selection. Intervention priorities should additionally consider severity, modifiability, feasibility, participant preferences, and independent evidence of treatment effectiveness. Coordination among academic, sport-support, and mental-health personnel may be useful when concerns cross physical, academic/performance-related, affective, and cognitive domains (Moreland et al., 2018; Young et al., 2023; Chang et al., 2020).

Several limitations should be emphasized. First, all participants were full-time students aged 18–22 years in sport-related majors at a single Chinese university. The observed network may reflect the cultural, educational, organizational, and sport-participation context of this institution and should not be directly generalized to other Chinese universities, different collegiate sport systems, elite or professional athletes, non-sport students, or other cultural settings.

Second, the primary analyses used a retained balanced panel. Complete pre-cleaning records for the 12 excluded participants are no longer reconstructable, preventing exact calculation of original-cohort prompt-level completion, formal comparison of retained and excluded participants, or an all-valid-observation sensitivity analysis. Participant exclusion may therefore have been informative, and bootstrap stability within the retained 60-person panel cannot eliminate this source of selection bias.

Third, the study used brief, study-specific single-item EMA indicators. Such items cannot represent the full multidimensional breadth of fatigue, pain, academic stress, performance worry, depression, affective arousal, rumination, concentration difficulty, or sleep quality, and item-level internal consistency cannot be evaluated. Measurement error may attenuate or otherwise influence conditional associations and centrality rankings. In particular, Q6 combined tension, anxiety, and irritability, so its network position cannot be assigned specifically to one component. Future studies should evaluate validated multi-item or multiple-indicator EMA measures while balancing psychometric breadth against repeated-assessment burden.

Fourth, the primary temporal network pooled two unequal within-day intervals of approximately 5.92 and 8.08 h. Although the broad outgoing pattern replicated across transition-specific analyses, several associations differed by transition, and mean levels varied systematically across the day. The exploratory overnight structure was markedly different from the within-day network. The primary temporal results should therefore be viewed as average conditional relations across the two sampled within-day transitions rather than as time-invariant dynamics.

Fifth, measured context influenced several momentary states and some temporal associations. Context-adjusted analyses supported the broad outgoing and contemporaneous pattern but showed substantial sensitivity of temporal in-strength and several academic-stress-related relations. Unmeasured contextual factors may likewise contribute to the observed network.

Sixth, the network and sleep analyses are observational. Directed temporal edges and prospective sleep coefficients establish measurement order but do not rule out unmeasured time-varying confounding, omitted variables, or reciprocal processes at other timescales. Centrality and bridge-centrality estimates describe structural position, not causal priority, clinical importance, or intervention effectiveness. Finally, the 14-day period may not capture slower cyclical processes that would emerge over longer monitoring windows.

Despite these limitations, the study contributes a more transparent intensive-longitudinal description of how specific physical, academic/performance-related, affective, and cognitive indicators are conditionally organized in daily life among students in sport-related majors. The combination of revised model specification, participant-level bootstrap accuracy, centrality stability analyses, transition- and context-sensitivity analyses, and prior-outcome-adjusted sleep models provides a more cautious basis for identifying recurring patterns worthy of further study.

## 5 Conclusion

Within the retained sample of students enrolled in sport-related majors at one Chinese university, the measured physical, academic/performance-related, affective, and cognitive states showed a structured pattern of within-day and same-prompt conditional associations. Soreness and fatigue occupied prominent outgoing positions in the temporal network, whereas the combined tension/anxiety/irritability indicator and rumination showed prominent contemporaneous and bridging positions. Some temporal relations, particularly those involving academic stress, were sensitive to training, academic-event, and time-of-day context. Poorer-than-usual sleep was prospectively associated with higher next-morning levels across all measured states after adjustment for previous-evening outcomes. These findings characterize the organization of the specific momentary indicators assessed in this sample and should not be interpreted as establishing a universal dual-career psychological system, causal pathways, or optimal intervention targets.

## Data Availability

The raw data supporting the conclusions of this article will be made available by the authors, without undue reservation.
