# Supplementary material for: Daily academic and sport-related strain and mental-health-related states among university students in sport-related majors: a 14-day ecological momentary assessment network study
Source: Front Psychol. 2026 Sep 7;17:1889946. doi: 10.3389/fpsyg.2026.1889946 (PMC13590340; doi:10.3389/fpsyg.2026.1889946)
Supplement: Supplementary file 1 [file Data_Sheet_1.pdf]

Supplementary Material

Note. This file contains the supplementary tables and figures referenced in the revised manuscript. The EMA instrument was administered in Chinese. English translations are provided for transparent reporting. Numerical results correspond to the final fixed-temporal specification unless otherwise noted.

Supplementary Table S1. Complete EMA instrument

Code	Analytic label	Domain / analytic role	Original Chinese item	English translation	Response anchors	Frequency
Q1	Fatigue	Physical Burden	此刻，我感到身体非常疲劳。	Right now, I feel very physically fatigued.	0 = not at all; 100 = extremely strong	Morning, midday, evening
Q2	Soreness / bodily pain	Physical Burden	此刻，我感到明显的肌肉酸痛或身体疼痛。	Right now, I feel noticeable muscle soreness or bodily pain.	0 = not at all; 100 = extremely strong	Morning, midday, evening
Q3	Academic stress	Academic / Performance Strain	此刻，我对我的学业进度或考试感到有压力。	Right now, I feel stressed about my academic progress or examinations.	0 = not at all; 100 = extremely strong	Morning, midday, evening
Q4	Performance worry	Academic / Performance Strain	此刻，我对我的运动训练表现或未来的比赛感到担忧。	Right now, I feel worried about my training performance or future competitions.	0 = not at all; 100 = extremely strong	Morning, midday, evening
Q5	Depressed mood	Affective Distress	此刻，我感到情绪低落或沮丧。	Right now, I feel low or depressed.	0 = not at all; 100 = extremely strong	Morning, midday, evening
Q6	Anxiety / Irritability	Affective Distress	此刻，我感到紧张、焦虑或烦躁。	Right now, I feel tense, anxious, or irritable.	0 = not at all; 100 = extremely strong	Morning, midday, evening
Q7	Rumination	Cognitive Difficulty	此刻，我脑海中反复盘旋着消极的想法（停不下来）。	Right now, negative thoughts keep recurring in my mind and I cannot stop them.	0 = not at all; 100 = extremely strong	Morning, midday, evening
Q8	Concentration difficulty	Cognitive Difficulty	此刻，我感觉很难集中注意力。	Right now, I find it difficult to concentrate.	0 = not at all; 100 = extremely strong	Morning, midday, evening
Q9	Poor sleep (previous night)	Additional sleep model	昨晚我的睡眠质量很差。	My sleep quality last night was poor.	0 = completely disagree; 100 = completely agree	Morning only

Interpretive note. Q1–Q8 are brief, study-specific momentary indicators and are not treated as validated single-item substitutes for multidimensional clinical scales. Q6 combines tension, anxiety, and irritability and is interpreted as a broad negative-affective-arousal indicator. Higher Q9 scores indicate poorer perceived previous-night sleep quality.

Supplementary Table S2. Elapsed-time distributions for observed EMA transitions

Transition	n	Mean (h)	SD	Median	Min	Max
Morning → Midday	840	5.917	0.000	5.917	5.917	5.917
Midday → Evening	840	8.083	0.247	7.900	7.900	8.417
Evening → Next morning	780	9.998	0.245	10.200	9.533	10.200

Note. Morning-to-midday and midday-to-evening transitions formed the primary within-day lag structure. Evening-to-next-morning transitions were excluded from the primary temporal network and analyzed exploratorily.

Supplementary Table S3. Transition-specific temporal sensitivity analyses

Panel A. Sample sizes by transition

Transition	Pairs	Rows	Participants
Morning → Midday	840	1680	60
Midday → Evening	840	1680	60
Evening → Next morning	780	1560	60

Panel B. Similarity of complete temporal coefficient matrices

Comparison	Pearson r	Spearman ρ	MAD	Sign agreement
Primary pooled vs Morning → Midday	0.734	0.634	0.177	73.2%
Primary pooled vs Midday → Evening	0.804	0.721	0.161	82.1%
Primary pooled vs Evening → Next morning	0.112	0.169	0.331	60.7%
Morning → Midday vs Midday → Evening	0.510	0.297	0.235	62.5%
Morning → Midday vs Evening → Next morning	0.057	0.064	0.324	55.4%
Midday → Evening vs Evening → Next morning	0.290	0.288	0.303	57.1%

Panel C. Temporal out-strength and in-strength by transition

Indicator	Transition	Out-strength	In-strength
Academic Stress	Primary pooled within-day	1.622	2.454
Anxiety / Irritability	Primary pooled within-day	1.010	3.257
Concentration Difficulty	Primary pooled within-day	1.076	1.127
Depressed Mood	Primary pooled within-day	1.159	1.289
Fatigue	Primary pooled within-day	2.867	1.630
Performance Worry	Primary pooled within-day	1.131	2.142
Rumination	Primary pooled within-day	1.779	1.536
Soreness	Primary pooled within-day	3.960	1.168
Academic Stress	Morning → Midday	1.277	0.754
Anxiety / Irritability	Morning → Midday	1.183	2.260
Concentration Difficulty	Morning → Midday	0.798	1.663
Depressed Mood	Morning → Midday	1.495	2.246
Fatigue	Morning → Midday	2.549	1.295
Performance Worry	Morning → Midday	0.736	2.044
Rumination	Morning → Midday	1.215	1.748
Soreness	Morning → Midday	3.870	1.115

Continued on next page

Indicator	Transition	Out-strength	In-strength
Academic Stress	Midday → Evening	2.057	0.541
Anxiety / Irritability	Midday → Evening	1.346	2.997
Concentration Difficulty	Midday → Evening	1.260	2.834
Depressed Mood	Midday → Evening	0.561	1.729
Fatigue	Midday → Evening	2.768	1.078
Performance Worry	Midday → Evening	1.330	2.038
Rumination	Midday → Evening	1.190	2.081
Soreness	Midday → Evening	3.979	1.192
Academic Stress	Evening → Next morning	1.474	1.237
Anxiety / Irritability	Evening → Next morning	2.201	1.700
Concentration Difficulty	Evening → Next morning	1.002	1.493
Depressed Mood	Evening → Next morning	0.951	1.921
Fatigue	Evening → Next morning	1.253	1.024
Performance Worry	Evening → Next morning	3.109	0.764
Rumination	Evening → Next morning	0.548	2.506
Soreness	Evening → Next morning	0.853	0.745

Note. Strength values are descriptive node-level summaries for each separately estimated temporal matrix. The overnight matrix is exploratory and is not treated as exchangeable with the primary within-day model.

Supplementary Table S4. Time-of-day mixed-effects results

Indicator	Contrast	Estimate	SE	95% CI	p	n
Fatigue	Midday vs Morning	8.01	0.37	[7.28, 8.74]	$< .001$	2520
Fatigue	Evening vs Morning	18.00	0.37	[17.27, 18.73]	$< .001$	2520
Soreness	Midday vs Morning	5.00	0.34	[4.33, 5.67]	$< .001$	2520
Soreness	Evening vs Morning	12.01	0.34	[11.34, 12.68]	$< .001$	2520
Academic Stress	Midday vs Morning	6.23	0.52	[5.22, 7.25]	$< .001$	2520
Academic Stress	Evening vs Morning	-2.01	0.52	[-3.02, -0.99]	$< .001$	2520
Performance Worry	Midday vs Morning	3.00	0.41	[2.19, 3.81]	$< .001$	2520
Performance Worry	Evening vs Morning	4.99	0.41	[4.18, 5.80]	$< .001$	2520
Depressed Mood	Midday vs Morning	3.53	0.25	[3.04, 4.01]	$< .001$	2520
Depressed Mood	Evening vs Morning	7.26	0.25	[6.77, 7.74]	$< .001$	2520
Anxiety / Irritability	Midday vs Morning	4.93	0.33	[4.28, 5.58]	$< .001$	2520
Anxiety / Irritability	Evening vs Morning	4.56	0.33	[3.91, 5.21]	$< .001$	2520
Rumination	Midday vs Morning	6.40	0.28	[5.86, 6.93]	$< .001$	2520
Rumination	Evening vs Morning	9.25	0.28	[8.71, 9.79]	$< .001$	2520
Concentration Difficulty	Midday vs Morning	5.93	0.21	[5.53, 6.33]	$< .001$	2520
Concentration Difficulty	Evening vs Morning	9.91	0.21	[9.51, 10.31]	$< .001$	2520

Note. Each model included assessment period, study day, and a participant random intercept. Morning was the reference assessment period. All models used 2,520 observations from 60 participants and were non-singular.

Supplementary Table S5. Contextual-variable sensitivity analyses

Panel A. Distribution and coding of training/competition status

Category	n	Proportion
No formal training / recovery-rest	324	12.9%
Routine training	1836	72.9%
Competition / selection / high-intensity competitive context	360	14.3%

Panel B. Distribution and coding of academic-event status

Category	n	Proportion
No salient academic event	450	17.9%
Routine academic activity	990	39.3%
Elevated academic task / preparation demand	720	28.6%
Examination / quiz context	360	14.3%

Panel C. Context-category fixed effects from mixed-effects models

Outcome	Contrast	Estimate	SE	95% CI	p	n
Fatigue	Routine training vs no formal training/recovery-rest	15.21	0.43	[14.37, 16.05]	$< .001$	2520
Fatigue	Competition/selection/high-intensity vs no formal training/recovery-rest	23.11	0.37	[22.38, 23.84]	$< .001$	2520
Fatigue	Routine academic activity vs no salient academic event	2.34	0.39	[1.58, 3.10]	$< .001$	2520
Fatigue	Elevated task/preparation demand vs no salient academic event	2.81	0.37	[2.09, 3.54]	$< .001$	2520
Fatigue	Examination/quiz vs no salient academic event	3.61	0.42	[2.80, 4.43]	$< .001$	2520
Soreness	Routine training vs no formal training/recovery-rest	13.92	0.43	[13.08, 14.76]	$< .001$	2520
Soreness	Competition/selection/high-intensity vs no formal training/recovery-rest	20.61	0.37	[19.89, 21.34]	$< .001$	2520
Soreness	Routine academic activity vs no salient academic event	2.49	0.39	[1.73, 3.24]	$< .001$	2520
Soreness	Elevated task/preparation demand vs no salient academic event	2.78	0.37	[2.06, 3.51]	$< .001$	2520
Soreness	Examination/quiz vs no salient academic event	3.28	0.41	[2.47, 4.09]	$< .001$	2520
Academic Stress	Routine training vs no formal training/recovery-rest	5.09	0.52	[4.07, 6.11]	$< .001$	2520
Academic Stress	Competition/selection/high-intensity vs no formal training/recovery-rest	-1.08	0.45	[-1.96, -0.19]	.017	2520
Academic Stress	Routine academic activity vs no salient academic event	3.44	0.47	[2.52, 4.37]	$< .001$	2520
Academic Stress	Elevated task/preparation demand vs no salient academic event	12.95	0.45	[12.07, 13.83]	$< .001$	2520
Academic Stress	Examination/quiz vs no salient academic event	27.01	0.50	[26.02, 27.99]	$< .001$	2520
Performance Worry	Routine training vs no formal training/recovery-rest	12.95	0.41	[12.14, 13.76]	$< .001$	2520
Performance Worry	Competition/selection/high-intensity vs no formal training/recovery-rest	28.85	0.36	[28.15, 29.55]	$< .001$	2520
Performance Worry	Routine academic activity vs no salient academic event	1.15	0.37	[0.42, 1.88]	.002	2520
Performance Worry	Elevated task/preparation demand vs no salient academic event	1.17	0.36	[0.48, 1.87]	$< .001$	2520
Performance Worry	Examination/quiz vs no salient academic event	1.10	0.40	[0.32, 1.88]	.006	2520
Depressed Mood	Routine training vs no formal training/recovery-rest	9.35	0.37	[8.62, 10.08]	$< .001$	2520
Depressed Mood	Competition/selection/high-intensity vs no formal training/recovery-rest	10.68	0.32	[10.05, 11.32]	$< .001$	2520
Depressed Mood	Routine academic activity vs no salient academic event	1.93	0.34	[1.27, 2.59]	$< .001$	2520
Depressed Mood	Elevated task/preparation demand vs no salient academic event	3.21	0.32	[2.58, 3.83]	$< .001$	2520
Depressed Mood	Examination/quiz vs no salient academic event	4.78	0.36	[4.08, 5.49]	$< .001$	2520
Anxiety / Irritability	Routine training vs no formal training/recovery-rest	10.09	0.40	[9.31, 10.87]	$< .001$	2520
Anxiety / Irritability	Competition/selection/high-intensity vs no formal training/recovery-rest	11.64	0.35	[10.96, 12.31]	$< .001$	2520

Continued on next page

Outcome	Contrast	Estimate	SE	95% CI	p	n
Anxiety / Irritability	Routine academic activity vs no salient academic event	2.79	0.36	[2.09, 3.50]	$< .001$	2520
Anxiety / Irritability	Elevated task/preparation demand vs no salient academic event	6.43	0.34	[5.76, 7.10]	$< .001$	2520
Anxiety / Irritability	Examination/quiz vs no salient academic event	11.80	0.39	[11.04, 12.56]	$< .001$	2520
Rumination	Routine training vs no formal training/recovery-rest	6.73	0.41	[5.92, 7.54]	$< .001$	2520
Rumination	Competition/selection/high-intensity vs no formal training/recovery-rest	9.28	0.36	[8.58, 9.98]	$< .001$	2520
Rumination	Routine academic activity vs no salient academic event	1.82	0.37	[1.09, 2.55]	$< .001$	2520
Rumination	Elevated task/preparation demand vs no salient academic event	4.95	0.36	[4.25, 5.65]	$< .001$	2520
Rumination	Examination/quiz vs no salient academic event	9.55	0.40	[8.77, 10.33]	$< .001$	2520
Concentration Difficulty	Routine training vs no formal training/recovery-rest	6.41	0.35	[5.73, 7.09]	$< .001$	2520
Concentration Difficulty	Competition/selection/high-intensity vs no formal training/recovery-rest	8.83	0.30	[8.24, 9.42]	$< .001$	2520
Concentration Difficulty	Routine academic activity vs no salient academic event	1.18	0.31	[0.56, 1.79]	$< .001$	2520
Concentration Difficulty	Elevated task/preparation demand vs no salient academic event	2.49	0.30	[1.90, 3.08]	$< .001$	2520
Concentration Difficulty	Examination/quiz vs no salient academic event	4.30	0.34	[3.65, 4.96]	$< .001$	2520

Note. Models additionally included assessment period, study day, and participant random intercepts. Category effects are relative to the stated reference context.

Panel D. Similarity of primary and context-adjusted network matrices

Comparison	Pearson r	Spearman ρ	MAD	Sign agreement
Primary vs context-residualized temporal	0.810	0.726	0.151	82.1%
Primary vs context-residualized contemporaneous	0.894	0.823	0.102	82.1%

Panel E. Similarity of primary and context-adjusted centrality rankings

Metric	Pearson r	Spearman ρ
Temporal out-strength	0.993	0.857
Temporal in-strength	0.136	0.357
Contemporaneous expected influence	0.965	0.976
Bridge expected influence	0.892	0.857

Panel F. Largest context-related temporal edge changes

From	To	Primary	Adjusted	Absolute change	Same sign
Academic Stress	Anxiety / Irritability	0.803	0.176	0.628	Yes
Rumination	Academic Stress	-0.612	-0.000	0.612	Yes
Fatigue	Academic Stress	-0.401	0.133	0.534	No
Concentration Difficulty	Academic Stress	-0.499	-0.016	0.483	Yes
Academic Stress	Rumination	0.220	-0.168	0.388	No
Performance Worry	Academic Stress	0.348	-0.034	0.381	No

Panel G. Largest context-related contemporaneous edge changes

From	To	Primary	Adjusted	Absolute change	Same sign
Academic Stress	Concentration Difficulty	-0.231	0.024	0.255	No
Academic Stress	Anxiety / Irritability	0.646	0.414	0.233	Yes
Fatigue	Academic Stress	-0.135	0.075	0.210	No
Soreness	Academic Stress	0.080	-0.129	0.209	No
Fatigue	Concentration Difficulty	0.456	0.250	0.207	Yes
Fatigue	Rumination	0.148	-0.038	0.187	No

Supplementary Table S6. Complete network coefficients with participant-level bootstrap 95% intervals

Panel A. Complete temporal coefficients

From	To	Estimate	Bootstrap 95% CI	Bootstrap mean	Bootstrap n
Fatigue	Fatigue	0.142	[0.103, 0.183]	0.143	1000
Fatigue	Soreness	0.949	[0.879, 1.038]	0.954	1000
Fatigue	Academic Stress	-0.401	[-0.482, -0.321]	-0.403	1000
Fatigue	Performance Worry	-0.703	[-0.780, -0.635]	-0.704	1000
Fatigue	Depressed Mood	-0.033	[-0.128, 0.062]	-0.035	1000
Fatigue	Anxiety / Irritability	-0.342	[-0.469, -0.224]	-0.345	1000
Fatigue	Rumination	-0.238	[-0.349, -0.141]	-0.241	1000
Fatigue	Concentration Difficulty	0.202	[0.113, 0.291]	0.204	1000
Soreness	Fatigue	0.706	[0.649, 0.769]	0.704	1000
Soreness	Soreness	-0.058	[-0.071, -0.045]	-0.058	1000
Soreness	Academic Stress	0.123	[0.084, 0.158]	0.123	1000
Soreness	Performance Worry	1.169	[1.080, 1.254]	1.164	1000
Soreness	Depressed Mood	0.503	[0.389, 0.627]	0.504	1000
Soreness	Anxiety / Irritability	0.505	[0.380, 0.632]	0.504	1000
Soreness	Rumination	0.482	[0.383, 0.588]	0.482	1000
Soreness	Concentration Difficulty	0.472	[0.355, 0.588]	0.471	1000
Academic Stress	Fatigue	-0.212	[-0.251, -0.172]	-0.211	1000
Academic Stress	Soreness	-0.065	[-0.094, -0.037]	-0.064	1000
Academic Stress	Academic Stress	1.134	[1.068, 1.199]	1.134	1000
Academic Stress	Performance Worry	0.038	[-0.005, 0.080]	0.037	1000
Academic Stress	Depressed Mood	-0.083	[-0.122, -0.045]	-0.083	1000
Academic Stress	Anxiety / Irritability	0.803	[0.732, 0.880]	0.805	1000
Academic Stress	Rumination	0.220	[0.168, 0.277]	0.219	1000
Academic Stress	Concentration Difficulty	0.202	[0.150, 0.262]	0.204	1000
Performance Worry	Fatigue	-0.072	[-0.096, -0.051]	-0.073	1000
Performance Worry	Soreness	-0.005	[-0.021, 0.014]	-0.004	1000
Performance Worry	Academic Stress	0.348	[0.300, 0.402]	0.350	1000
Performance Worry	Performance Worry	0.765	[0.735, 0.796]	0.765	1000
Performance Worry	Depressed Mood	-0.058	[-0.092, -0.026]	-0.059	1000
Performance Worry	Anxiety / Irritability	0.457	[0.409, 0.507]	0.459	1000
Performance Worry	Rumination	0.123	[0.090, 0.156]	0.123	1000

Continued on next page

From	To	Estimate	Bootstrap 95% CI	Bootstrap mean	Bootstrap n
Performance Worry	Concentration Difficulty	0.068	[0.037, 0.100]	0.068	1000
Depressed Mood	Fatigue	-0.089	[-0.126, -0.054]	-0.089	1000
Depressed Mood	Soreness	-0.005	[-0.029, 0.021]	-0.005	1000
Depressed Mood	Academic Stress	0.386	[0.322, 0.453]	0.386	1000
Depressed Mood	Performance Worry	-0.053	[-0.097, -0.009]	-0.052	1000
Depressed Mood	Depressed Mood	0.001	[-0.035, 0.038]	0.002	1000
Depressed Mood	Anxiety / Irritability	0.427	[0.384, 0.472]	0.428	1000
Depressed Mood	Rumination	-0.126	[-0.178, -0.080]	-0.126	1000
Depressed Mood	Concentration Difficulty	-0.073	[-0.114, -0.030]	-0.073	1000
Anxiety / Irritability	Fatigue	0.090	[0.050, 0.130]	0.091	1000
Anxiety / Irritability	Soreness	0.077	[0.044, 0.109]	0.076	1000
Anxiety / Irritability	Academic Stress	0.086	[0.019, 0.157]	0.086	1000
Anxiety / Irritability	Performance Worry	-0.085	[-0.129, -0.042]	-0.085	1000
Anxiety / Irritability	Depressed Mood	0.285	[0.250, 0.321]	0.286	1000
Anxiety / Irritability	Anxiety / Irritability	-0.019	[-0.079, 0.039]	-0.020	1000
Anxiety / Irritability	Rumination	0.325	[0.271, 0.386]	0.326	1000
Anxiety / Irritability	Concentration Difficulty	0.062	[0.025, 0.102]	0.062	1000
Rumination	Fatigue	0.294	[0.253, 0.335]	0.294	1000
Rumination	Soreness	0.047	[0.018, 0.078]	0.046	1000
Rumination	Academic Stress	-0.612	[-0.673, -0.551]	-0.612	1000
Rumination	Performance Worry	-0.054	[-0.089, -0.016]	-0.054	1000
Rumination	Depressed Mood	0.184	[0.141, 0.227]	0.184	1000
Rumination	Anxiety / Irritability	-0.539	[-0.578, -0.497]	-0.540	1000
Rumination	Rumination	0.016	[-0.041, 0.075]	0.016	1000
Rumination	Concentration Difficulty	-0.049	[-0.093, -0.012]	-0.051	1000
Concentration Difficulty	Fatigue	0.167	[0.133, 0.200]	0.166	1000
Concentration Difficulty	Soreness	-0.022	[-0.036, -0.007]	-0.022	1000
Concentration Difficulty	Academic Stress	-0.499	[-0.582, -0.430]	-0.498	1000
Concentration Difficulty	Performance Worry	0.040	[0.002, 0.079]	0.040	1000
Concentration Difficulty	Depressed Mood	0.142	[0.117, 0.171]	0.142	1000
Concentration Difficulty	Anxiety / Irritability	-0.183	[-0.236, -0.132]	-0.183	1000
Concentration Difficulty	Rumination	0.023	[-0.028, 0.071]	0.023	1000
Concentration Difficulty	Concentration Difficulty	-0.029	[-0.061, -0.001]	-0.030	1000

Note. Rows include autoregressive self-loops and all cross-lagged coefficients from the final fixed-temporal model. Temporal coefficients are standardized conditional regression coefficients.

Panel B. Complete contemporaneous coefficients

From	To	Estimate	Bootstrap 95% CI	Bootstrap mean	Bootstrap n
Fatigue	Soreness	0.387	[0.336, 0.433]	0.387	1000
Fatigue	Academic Stress	-0.135	[-0.179, -0.095]	-0.138	1000
Fatigue	Performance Worry	-0.022	[-0.065, 0.022]	-0.021	1000
Fatigue	Depressed Mood	0.177	[0.115, 0.237]	0.176	1000
Fatigue	Anxiety / Irritability	-0.155	[-0.207, -0.104]	-0.155	1000
Fatigue	Rumination	0.148	[0.102, 0.194]	0.148	1000
Fatigue	Concentration Difficulty	0.456	[0.417, 0.484]	0.454	1000
Soreness	Academic Stress	0.080	[0.049, 0.115]	0.081	1000
Soreness	Performance Worry	0.041	[0.005, 0.079]	0.041	1000
Soreness	Depressed Mood	0.149	[0.104, 0.198]	0.149	1000
Soreness	Anxiety / Irritability	0.017	[-0.028, 0.062]	0.017	1000
Soreness	Rumination	-0.170	[-0.201, -0.142]	-0.169	1000
Soreness	Concentration Difficulty	-0.102	[-0.142, -0.060]	-0.101	1000
Academic Stress	Performance Worry	-0.378	[-0.408, -0.341]	-0.375	1000
Academic Stress	Depressed Mood	-0.207	[-0.256, -0.147]	-0.204	1000
Academic Stress	Anxiety / Irritability	0.646	[0.622, 0.666]	0.644	1000
Academic Stress	Rumination	0.440	[0.398, 0.476]	0.437	1000
Academic Stress	Concentration Difficulty	-0.231	[-0.275, -0.182]	-0.229	1000
Performance Worry	Depressed Mood	-0.185	[-0.242, -0.121]	-0.182	1000
Performance Worry	Anxiety / Irritability	0.313	[0.269, 0.351]	0.310	1000
Performance Worry	Rumination	0.279	[0.230, 0.326]	0.278	1000
Performance Worry	Concentration Difficulty	-0.114	[-0.167, -0.058]	-0.112	1000
Depressed Mood	Anxiety / Irritability	0.372	[0.331, 0.410]	0.369	1000
Depressed Mood	Rumination	0.470	[0.419, 0.520]	0.468	1000
Depressed Mood	Concentration Difficulty	-0.200	[-0.226, -0.176]	-0.200	1000
Anxiety / Irritability	Rumination	-0.205	[-0.262, -0.143]	-0.202	1000
Anxiety / Irritability	Concentration Difficulty	0.509	[0.456, 0.553]	0.505	1000
Rumination	Concentration Difficulty	-0.022	[-0.050, 0.008]	-0.021	1000

Note. Contemporaneous edge weights are partial correlations from the within-person same-window Gaussian graphical-model layer.

Panel C. Complete between-person coefficients

From	To	Estimate	Bootstrap 95% CI	Bootstrap mean	Bootstrap n
Fatigue	Soreness	0.679	[0.526, 0.794]	0.677	1000
Fatigue	Academic Stress	0.065	[-0.209, 0.356]	0.078	1000
Fatigue	Performance Worry	-0.190	[-0.390, 0.032]	-0.188	1000
Fatigue	Depressed Mood	-0.016	[-0.277, 0.249]	-0.014	1000
Fatigue	Anxiety / Irritability	-0.029	[-0.343, 0.293]	-0.031	1000
Fatigue	Rumination	0.018	[-0.260, 0.306]	0.015	1000
Fatigue	Concentration Difficulty	0.065	[-0.185, 0.321]	0.064	1000
Soreness	Academic Stress	-0.157	[-0.407, 0.083]	-0.164	1000
Soreness	Performance Worry	0.579	[0.335, 0.788]	0.572	1000
Soreness	Depressed Mood	-0.036	[-0.275, 0.233]	-0.035	1000
Soreness	Anxiety / Irritability	0.199	[-0.030, 0.414]	0.200	1000
Soreness	Rumination	0.155	[-0.058, 0.372]	0.161	1000
Soreness	Concentration Difficulty	-0.069	[-0.255, 0.144]	-0.070	1000
Academic Stress	Performance Worry	-0.281	[-0.530, -0.002]	-0.283	1000
Academic Stress	Depressed Mood	0.435	[0.236, 0.618]	0.434	1000
Academic Stress	Anxiety / Irritability	0.208	[-0.057, 0.471]	0.208	1000
Academic Stress	Rumination	0.740	[0.588, 0.852]	0.737	1000
Academic Stress	Concentration Difficulty	-0.263	[-0.524, 0.002]	-0.263	1000
Performance Worry	Depressed Mood	0.310	[0.035, 0.566]	0.307	1000
Performance Worry	Anxiety / Irritability	-0.069	[-0.330, 0.224]	-0.068	1000
Performance Worry	Rumination	0.114	[-0.173, 0.372]	0.112	1000
Performance Worry	Concentration Difficulty	-0.019	[-0.321, 0.282]	-0.024	1000
Depressed Mood	Anxiety / Irritability	0.214	[-0.044, 0.456]	0.216	1000
Depressed Mood	Rumination	-0.020	[-0.292, 0.258]	-0.020	1000
Depressed Mood	Concentration Difficulty	0.348	[0.057, 0.567]	0.336	1000
Anxiety / Irritability	Rumination	-0.286	[-0.513, -0.063]	-0.300	1000
Anxiety / Irritability	Concentration Difficulty	0.481	[0.192, 0.756]	0.486	1000
Rumination	Concentration Difficulty	0.373	[0.140, 0.577]	0.379	1000

Note. Between-person edge weights are partial correlations among participant-level mean scores across the 14-day period. This layer is descriptive and showed comparatively wide bootstrap uncertainty.

Supplementary Figure S1. Participant-level bootstrap intervals for centrality indices

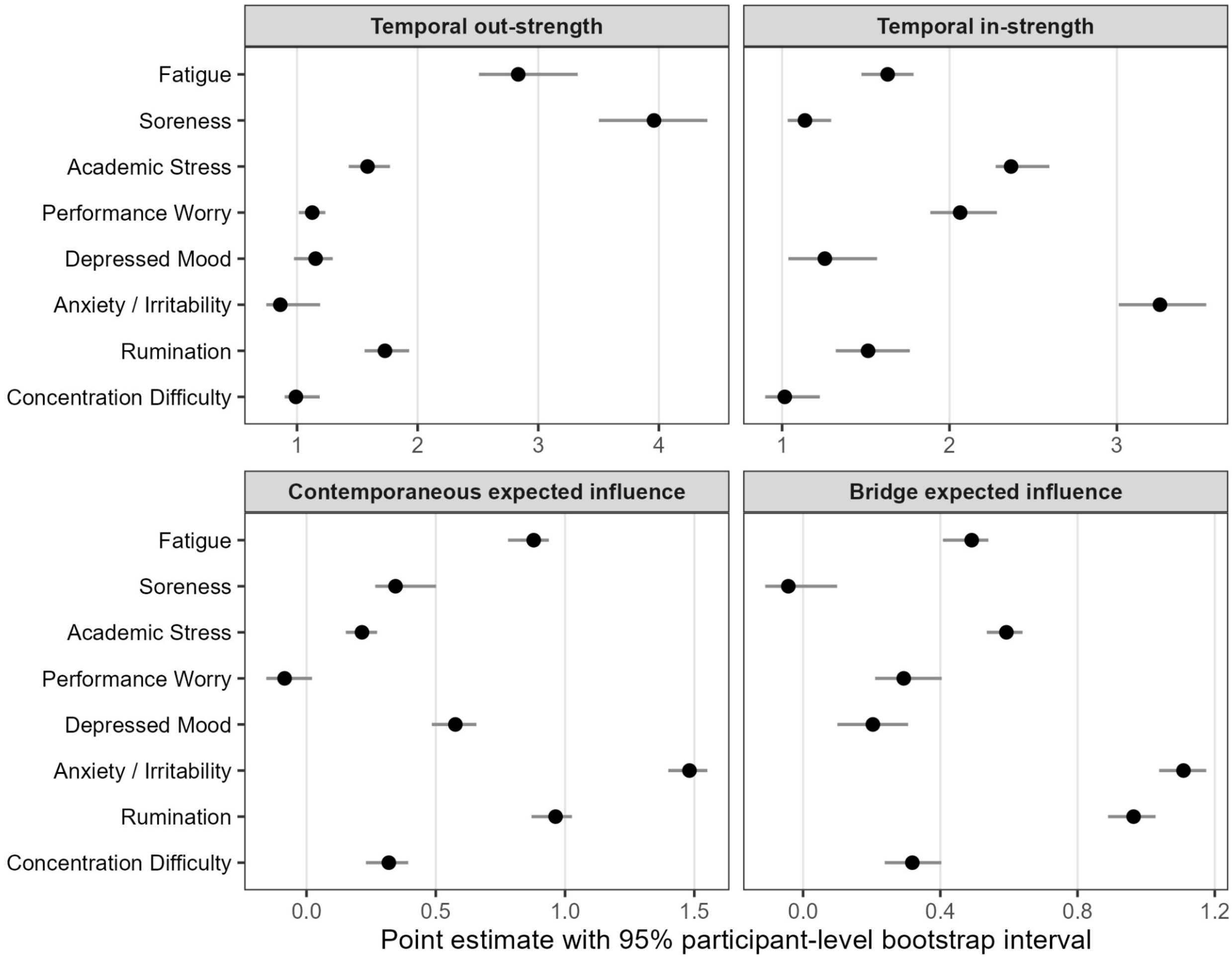

Caption. Points show full-sample centrality estimates and horizontal bars show participant-level bootstrap 95% intervals under the final fixed-temporal specification. Temporal out-strength and in-strength exclude autoregressive self-loops.

Supplementary Figure S2. Participant-dropping stability of centrality indices

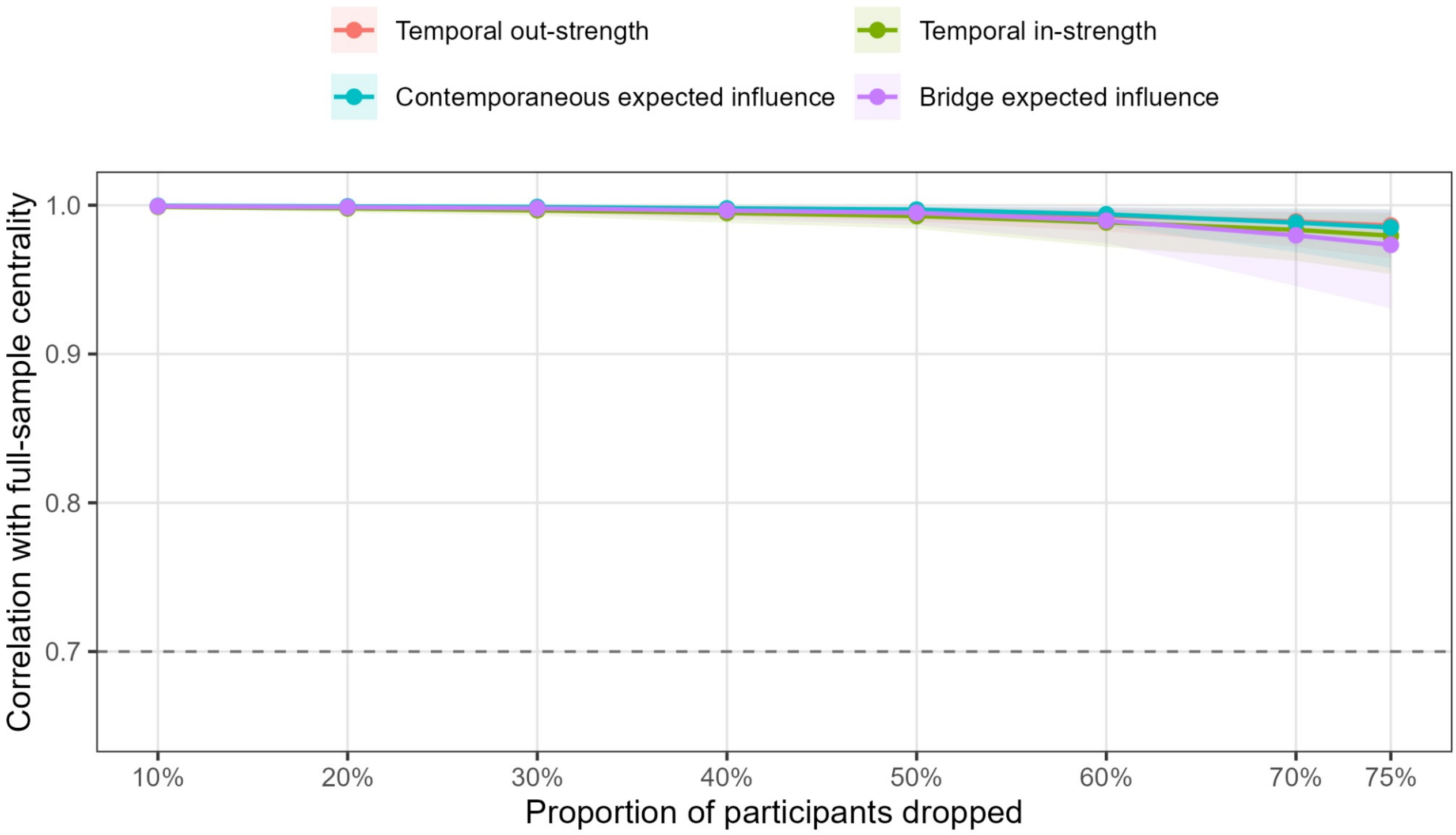

Caption. Curves summarize correlations between subset-based and full-sample centrality estimates across participant-deletion proportions. Points and ribbons summarize successfully estimated subset models. Under the conservative CS criterion, failed subset fits were treated as not satisfying the $r \geq .70$ stability requirement. Because only 188 of 200 models converged at 75% participant deletion, the final CS($r = .70$) coefficient was .70 for all four centrality metrics.
